# Successful Treatment of Left Ventricle Inferior Wall Perforation and Rupture Associated with an Impella 5.5: A Case Report

**DOI:** 10.3390/reports8020098

**Published:** 2025-06-19

**Authors:** James Hall, Hasnayn Raza, Sarah Lee, Nicole Bryce, Sunil Abrol

**Affiliations:** 1 Department of Cardiac Surgery, NYU Langone Long Island Hospital, Mineola, NY 11501, USA; sunil.abrol@nyulangone.org; 2 Department of Biology, State University of New York, Albany, NY 12246, USA; razahasnayn@gmail.com; 3 NYU Grossman Long Island School of Medicine, Mineola, NY 11501, USA; sarah.lee4@nyulangone.org (S.L.); nicole.bryce@nyulangone.org (N.B.)

**Keywords:** Impella, ventricular perforation, ECMO, free wall rupture, myocardial infarction

## Abstract

**Background and Clinical Significance:** Perforation of the left ventricle related to microaxial ventricular assist devices (Impella) is a rare but fatal complication related to placement or adjustment. It results in left ventricular hemorrhage and tamponade, leading to rapid deterioration and death. **Case Presentation:** We present a case report of a 73-year-old man who developed this complication postoperatively and was successfully managed to a full recovery. **Conclusions:** To our knowledge, he is the only reported patient to have this complication outside the setting of immediate placement who subsequently survived to discharge.

## 1. Introduction and Clinical Significance

Microaxial ventricular assist devices such as Impella (Abiomed, Danvers, MA, USA) are peripherally inserted intraventricular pumps that remove blood from the left ventricle and eject it into the ascending aorta, thereby off-loading the ventricle. They are indicated for support of reversible causes of cardiac failure, such as decompensations or exacerbations of chronic systolic failure and cardiac surgery, and for bridges to other advanced therapies, such as implantable left ventricular assist devices or cardiac transplants [1]. They are often used as a left ventricular vent during conditions of increased afterload created by peripheral veno-arterial extracorporeal membrane oxygenation (VA-ECMO). Despite failing to show any definitive mortality benefit in multiple studies, they have become a mainstay of short-term cardiac support. An estimated 20,000 to 25,000 Impellas are inserted in the United States every year [2]. Despite the suggested benefits and positive clinical experience, there have been several reports of left ventricular wall perforations associated with their use [3,4,5]. While this complication remains rare, reported by one registry to be 0.02%, it remains a significant concern, particularly in the setting of the weakening of the left ventricular (LV) wall that occurs in the week following acute infarction [6]. According to a report based on the FDA-MAUDE reporting database, 50 ventricular perforations associated with Impella devices were reported between 2009 and 2021. The majority of these occurred during device repositioning (24%), intraoperative cardiac manipulation (14%), or were related to guidewires (14%) [7].

The most common location in the heart for perforations is the apex, likely because of thinner myocardium and being directly in the path of the device. The second most common reported location is the anterolateral wall. Inferior locations have been reported intraoperatively with manipulation [7]. Many models of Impellas feature a flexible pigtail on the end designed to stabilize the device, but in higher flow models, such as the Impella 5.5, this has been removed to facilitate a higher inflow. This complication can be a particular concern in the setting of the weakening of the ventricular (LV) wall that occurs following acute infarction [8]. While rare, reported to be 0.02% by one registry, this complication has been reported to result in death 68% of the time from rapid hemorrhage and tamponade [8].

## 2. Case Presentation

**Figure 1:**
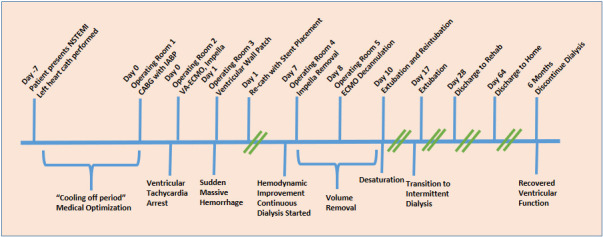
Timeline of events.

A seventy-three-year-old man with a past medical history of coronary artery disease, hypertension, and prostate cancer presented with shortness of breath, abdominal discomfort, diarrhea, and lower extremity edema and was found to have a non-ST elevation myocardial infarction (NSTEMI) complicated by acute systolic heart failure with a left ventricular ejection fraction of 20% and new severe mitral regurgitation. He underwent left heart catheterization, revealing severe three-vessel disease (complete right coronary occlusion, critical mid-left-anterior descending stenosis, and critical stenosis of the ostial circumflex artery) and an infarct covering the inferior wall of the left ventricle. The patient was admitted to the coronary care unit and treated with a heparin infusion and diuretics, while the cardiac surgery service was consulted for cardiac bypass surgery (CABG). Refer to the timeline in Figure 1.

On his seventh day following his infarction, the patient went to the operating room for a three-graft CABG and mitral valve annuloplasty. Given his poor function, an intra-aortic balloon pump (IABP) was placed for support. A few hours after coming to the intensive care unit (ICU), the patient developed refractory ventricular tachycardia requiring emergent cardioversion, pharmacologic treatment, and pacing. He returned to the operating room where the IABP was removed, and he was cannulated for peripheral veno-arterial (VA) extracorporeal membrane oxygenation (ECMO). An Impella 5.5 was inserted via the left subclavian artery for LV support. He returned to the ICU with an open chest and was stabilized hemodynamically throughout the night despite multiple additional episodes of tachycardia treated with amiodarone, magnesium, and lidocaine.

Early the following morning on postoperative day (POD) 1, the patient became somewhat agitated and attempted to sit up in bed. This precipitated a sudden large volume output of bright red blood from the patient’s chest tubes, draining approximately 1 L of blood within 20 min. On the basis of the suddenness and severity of this new bleed, a ventricular or aortic rupture was suspected. There were no delays for additional imaging or testing. Rapid blood transfusion was initiated, and he was emergently returned to the operating room, where he was found to have a frank rupture of the inferior wall of the left ventricle, the site of his recent infarct. A 10 cm × 6 cm felt and pericardial patch was sewn over the defect and entire inferior surface of the heart with resolution of active bleeding. It was felt that due to poor tissue integrity, the patch would need to be large. Upon return to the ICU, he continued to have poor LV contractility and underwent repeat left heart catheterization to assess the patency of his grafts, during which he received an additional drug-eluting stent. His renal function was severely impaired, and he required continuous dialysis through the ECMO circuit, but his hemodynamics improved over the following week. The Impella device was removed on POD 7. By POD 8, the LV ejection fraction had improved to 40%, and he returned to the operating room for ECMO decannulation. He was extubated but re-intubated on POD 10 and then re-extubated on POD 17. He transitioned to intermittent hemodialysis and was discharged to a rehabilitation center on POD 28. He spent 36 days in rehab and was able to return home. His renal function gradually improved, and dialysis was discontinued 6 months later. An echocardiogram one year later revealed moderate inferior hypokinesis of the LV, but an ejection fraction of 55%.

## 3. Discussion

Response to a ruptured ventricular wall is a complication that requires immediate recognition and intervention. Our patient benefited from having chest tubes already in place, a surgical team immediately available, and likely the time of day that this complication occurred. Without these factors, this patient likely would have suffered a poor outcome. In terms of downsides, it is possible, given his weakened LV wall, that a small device that featured a pigtail may have avoided this particular complication while still adequately venting the LV. This is uncertain, as perforations have still been noted to occur with this added safety measure. The rationale for utilizing the 5.5 model in this patient’s case was that following decannulation from VA-ECMO, it might be used independently for further ventricular support while awaiting the return of ventricular function.

In addition, while recent trends have included minimizing sedation and respiratory support and even facilitating ambulation, these normalizing improvements should not obscure the importance of preventing agitation or excessive movement with invasive medical devices in place.

Of the published case reports, only one patient was noted to have survived a non-placement-related free wall rupture associated with a device, and this patient unfortunately later succumbed to sepsis. Most of the patients who survive these ventricular wall perforations suffer them during placement or manipulation, including smaller perforations by guidewires. These are recognized immediately with often already open chests, facilitating repair [3,4].

Given the low frequency—high consequence nature of this complication, additional caution should be warranted in certain settings, including patients with infarct-weakened ventricular walls. It may be reasonable to consider alternative support, if available. The microaxial ventricular assist device remains a mainstay of mechanical cardiac support, and while ventricular wall perforation is rare, constant surveillance is required. Particular attention should be paid to all during transitions, transfers, and episodes of agitation to avoid malpositioning or traumatic events.

## 4. Conclusions

We report an instance of this rare but fatal complication with a uniquely good outcome, highlighting the need for care in choosing devices, vigilance in monitoring their use, and that in the setting of unexpected ventricular wall perforations, positive outcomes are possible with aggressive intervention.

## Data Availability

The original contributions presented in this study are included in the article. Further inquiries can be directed to the corresponding author.

## Abbreviations

The following abbreviations are used in this manuscript:

CABG	Coronary Artery Bypass Graft
ECMO	Extra Corporeal Membrane Oxygenation
IABP	Intra-Arterial Balloon Pump
ICU	Intensive Care Unit
LV	Left Ventricle
NSTEMI	Non-ST Elevation Myocardial Infarction
POD	Postoperative Day
